# Residence in High-Crime Neighborhoods Moderates the Association Between Interleukin 6 and Social and Nonsocial Reward Brain Responses

**DOI:** 10.1016/j.bpsgos.2022.04.006

**Published:** 2022-05-18

**Authors:** Iris Ka-Yi Chat, Andrew A. Gepty, Marin Kautz, Naoise Mac Giollabhui, Zoe V. Adogli, Christopher L. Coe, Lyn Y. Abramson, Thomas M. Olino, Lauren B. Alloy

**Affiliations:** a Department of Psychology and Neuroscience, Temple University, Philadelphia, Pennsylvania; b Psychological and Brain Sciences Department, George Washington University, Washington, District of Columbia; c Depression Clinical Research Program, Massachusetts General Hospital, Boston, Massachusetts; d Department of Psychology, University of Wisconsin, Madison, Wisconsin

**Keywords:** fMRI, Inflammation, Neighborhood crime, Nucleus accumbens, Orbitofrontal cortex, Reward

## Abstract

**Background:**

Residence in high-crime neighborhoods, especially in childhood, is linked to mental health issues later. Detecting distinct neurobiological processes underlying the effects of this environmental stressor may be critical to identifying prevention and intervention targets. This study examined the relationships of levels of a circulating inflammatory protein with social and monetary reward–related brain function among adolescents who lived in high- versus low-crime neighborhoods during childhood.

**Methods:**

A total of 70 participants (mean age = 16.3 years; 57% female) completed measures of inflammatory markers, depression history, and health and 2 functional magnetic resonance imaging tasks assessing responsivity to monetary and social rewards. Multivariate linear regression tested whether individuals with higher interleukin 6, an inflammatory cytokine, who also lived in neighborhoods with higher crime had distinct orbitofrontal cortex and nucleus accumbens activation to monetary reward and social acceptance.

**Results:**

For adolescents who lived in neighborhoods with more crime, higher interleukin 6 was associated with higher nucleus accumbens responses to social acceptance. We did not detect significant moderating effects of neighborhood crime rates on the associations of interleukin 6 with orbitofrontal cortex responses to social acceptance or orbitofrontal cortex/nucleus accumbens activation during monetary reward anticipation or outcome. These results were obtained before and after adjusting for neighborhood income and other covariates. We did not detect significant moderating effects of neighborhood income.

**Conclusions:**

High-threat residence environment and specific demands of the social context in childhood may have shaped the effect of peripheral immune activation on reward-related neural function in adolescence. The prevailing view that inflammation-associated behaviors are characterized by blunted responsiveness to reward may be oversimplistic.

Living in a high-crime neighborhood is a major public health concern with risks to personal safety and negative health outcomes (1,2), especially if the adversity occurs in childhood (3). Identifying mechanisms underlying psychiatric risk is needed to design more effective interventions. Residence in high-crime neighborhoods early in life may shape neurobiological processes that contribute to psychological health, although specific pathways remain unclear (4, 5, 6, 7). Identifying specific neurobiological processes associated with childhood residence in high-crime neighborhoods may be key to identifying precise intervention targets tailored to this adversity. This study investigated the associations between inflammation and reward-related brain activation, a specific neurobiological process that may explain health-related consequences of high-crime neighborhood residence in childhood.

Neurobiological processes underlying motivation and reward function play substantial roles in the pathogenesis of psychiatric conditions (8, 9, 10). Motivation and reward function are linked to a corticostriatal neural circuit. The nucleus accumbens (NAc) and orbitofrontal cortex (OFC) are centrally involved in this circuit (11). The hedonic properties of rewarding stimuli are represented in the NAc, and the OFC is known for computation of reward value and reward-related decision making (11). Hypo/hyperactivation in these brain regions has been associated with behaviors that characterize psychiatric conditions, including anhedonia, elevated approach motivation, and impulsivity (9,10,12, 13, 14).

Accumulating research highlights the role of immune function, particularly inflammatory proteins, in signaling reward function (15, 16, 17, 18, 19). Inflammatory proteins can influence reward processing by accessing the brain via multiple direct and indirect pathways and altering reward-related dopaminergic tone and basal ganglia function (20,21). A widely accepted view posits that inflammation dampens reward responsivity (15,16,22,23). However, there are noted exceptions. Inflammation has been associated with elevated neural response to social reward (e.g., positive social feedback and viewing close others) (24,25) and blunted neural response to nonsocial reward (e.g., money) (26). Consistent with a context-dependence hypothesis positing that there are moderators of the pattern of relationships between inflammation and reward function (24,25,27, 28, 29, 30, 31, 32), these studies suggest that such relationships depend on type of reward. In sum, inflammation and its modulation of reward-related brain function together may be relevant neurobiological mechanisms underlying risk for psychiatric conditions. Furthermore, examining the complex pattern of immune-brain communication that depends on contextual factors may offer insight into negative health outcomes.

One context that influences the relationship between inflammatory proteins and reward function may be exposure to neighborhood crime. There are separate literatures on the associations between living in high-crime neighborhoods and immunity and neural functioning (33, 34, 35, 36). The neuroimmune network hypothesis posits that exposure to threatening environments in childhood may potentiate threat-related neural circuitry and shape and may sensitize immune cells in a manner that amplifies communication with the reward-related neural circuit and, thus, amplifies changes in motivation and behaviors that emerge later in adolescence and adulthood (19). Accordingly, exposure to high-crime neighborhoods during childhood may strengthen communication between circulating inflammatory proteins and the reward circuit (19). Specifically, inflammation could be related to less motivation for nonsocial reward, particularly for those from high-crime neighborhoods. Although the neuroimmune network hypothesis did not explicitly provide a prediction for social reward, converging with the context-dependence hypothesis, inflammation could be associated with more motivation toward social reward, especially for those from high-crime neighborhoods (19,37).

This study examined the relationship between inflammatory signaling and reward-related brain function among adolescents with varying crime rates in their childhood neighborhoods. We hypothesized that the association between inflammatory activity and reward function would be stronger at higher levels of childhood neighborhood crime rates. Specifically, higher peripheral cytokine levels, as measured by interleukin (IL) 6, would be associated with blunted neural responsiveness to monetary reward but elevated neural responsiveness to social reward (19,27,37). These associations would be stronger among individuals from high-crime neighborhoods in childhood. We focus on adolescence, a developmental period known for increased prevalence of first-onset psychiatric disorders. Adolescence also is characterized by normative maturation in immune function and rapid changes in reward-related neural circuitry (38, 39, 40). Thus, the neurobiological impact of childhood exposure to neighborhood crime may be pronounced during adolescence. Because high neighborhood crime rates often are accompanied by low neighborhood income, we adjusted for neighborhood income in the analyses and also examined the separate moderating effect of neighborhood income. Another study objective was to explore whether results differed by types of neighborhood crimes that vary in degree of threat (i.e., violent vs. nonviolent) (41).

To test these hypotheses, adolescents completed 2 functional magnetic resonance imaging (fMRI) tasks to assess social versus nonsocial (i.e., monetary) reward-related brain function and a blood draw to quantify circulating levels of inflammatory proteins. To avoid multiple comparisons, IL-6 was selected as the bioindicator of interest because this protein has been most reliably associated with stress (42,43). Exploratory analyses determined whether results were comparable using C-reactive protein (CRP), given the marker’s correlation with IL-6. Crime incidence data for the neighborhood in which each participant lived at study entry (approximately 36 months before the blood draw and fMRI) were extracted from open access crime statistics. These analyses may help to advance our understanding of the pathways that underlie the connections between environmental adversity and neuroimmune processes that influence reward function.

## Methods and Materials

### Participants

Participants were drawn from a longitudinal study of adolescent-onset depression (44) that recruited participants from middle schools in the Philadelphia area. All participants still involved in the parent study when the fMRI scan was introduced were invited except those with a current diagnosis of alcohol/substance use disorder, bipolar disorder, psychosis, pervasive developmental disorder, obsessive-compulsive disorder, or posttraumatic stress disorder; claustrophobia; ferrous metal in any part of the body; lifetime history of head trauma; left-handedness; or pregnancy. Of the 86 participants who completed the supplemental fMRI study, 16 were excluded owing to task acquisition issues (*n* = 2) or unavailability of crime statistics (*n* = 14). Thus, the final sample included 70 participants. This final sample did not differ from the imaging subset who were excluded in sex (χ^2^_1_ = 1.36, *p* = .24), race (χ^2^_2_ = 4.05, *p* = .13), age at blood draw (*t*_52_ = −0.329, *p* = .37), or body mass index (*t*_67_ = −0.139, *p* = .89). More individuals from the analytic sample received subsidized lunch (χ^2^_2_ = 6.99, *p* = .01).

### Procedures

Neighborhood crime and income were assessed at baseline (see the Supplement). Diagnostic interviews assessing depression history were given at baseline and annually throughout a participant’s enrollment (see the Supplement). Approximately 48 months after the launch of the longitudinal study, annual blood draws were introduced along with health measures (e.g., body mass index, prescription medication [psychotropic/anti-inflammatory] use, and major illness^1^). Participants who engaged in this study completed 2 fMRI tasks and a blood draw at the same assessment. Study procedures were approved by Temple University’s Institutional Review Board. Informed consent was obtained prior to participation.

### Measures

#### Peripheral Inflammatory Markers

Nonfasting blood was obtained in the late afternoon to control for diurnal variation in inflammatory physiology (45,46). From antecubital blood, plasma levels of IL-6, IL-8, IL-10, and tumor necrosis factor-α were quantified by multicytokine array, and high-sensitivity CRP was determined in a singleplex assay, using an electrochemiluminescence platform and a QuickPlex SQ 120 imager for quantification of cytokines and CRP (Meso Scale Discovery). Samples were assayed in duplicate, with intra-assay coefficients of variation between 1.94% and 4.38%, and values referenced to a standard curve generated from 7 calibrators with known concentrations. The lower limit of detection was 0.1 pg/mL, with a large dynamic range up to 2000 pg/mL. Values of the markers were log-transformed to normalize the distribution of values and bring them into an acceptable range of skewness.

#### Chatroom fMRI Paradigm

The chatroom interact task measures neural responses to social feedback from online peer interactions (47), with predictive criterion-related validity shown in adolescent samples with and without psychiatric conditions (48,49). During the setup, participants ranked virtual similar age- and sex-matched peers on how much they would like to interact with them based on a brief online profile (photograph and interests). During the scan, participants were told that 2 virtual peers and the participants would take turns choosing which one of them they would like to interact with on a specific topic (i.e., sports, food). In the first block, participants were told they had been randomly selected to choose first. In the second and third blocks, participants observed their 2 peers choosing between the participant and the other peer as a preferred interaction partner. These 2 trials were arranged in blocks with predominant (i.e., 70%) acceptance feedback and rejection feedback, respectively. During feedback, a gray box framing their photograph or a large gray X across their photograph, indicating whether the participant was chosen or not chosen, was displayed for 10 seconds. A final block, used as a motor and perceptual control, instructed the participant to identify which face (participant’s or peer’s) had a small gray dot on it.

#### Card Guessing fMRI Paradigm

The card guessing task examines brain activation during anticipation and outcome of monetary reward (50). Participants were instructed to press a button in 4 seconds to guess whether the value of a card is greater or less than 5. Then, an up arrow indicating a reward trial or down arrow indicating a loss trial was displayed for 6 seconds. On reward trials, participants won $1 if they made the correct guess, and they did not win money if they made a wrong guess or the number was 5. On loss trials, they avoided losing money if they made a correct guess or the number was 5, and they lost $0.50 if they made a wrong guess. Feedback about trial outcomes (i.e., win, break even, lose) was displayed for 5000 ms. Finally, a jittered fixation cross was presented for 9000 ms as an intertrial interval. The task had 24 trials and lasted approximately 9 minutes. The sequence of trial outcomes was predetermined to allow for a similar experience of rewarding outcomes in the same order for all participants. Participants were told that outcomes of each trial are the result of chance, given that striatal response to rewarding cues occurs in particular to unpredicted reward (51).

#### fMRI Acquisition and Analysis

Neuroimaging data were collected on a 3T Siemens MRI scanner. Blood oxygen level–dependent functional images were acquired with a gradient echo planar imaging sequence and covered 34 axial slices (3 mm thick) beginning at the cerebral vertex and encompassing the entire cerebrum and most of the cerebellum (repetition time = 2000 ms, echo time = 25 ms, field of view = 20 cm, matrix 64 × 64). Before data collection, an echo planar imaging scan was acquired for visual inspection of artifacts and signal level across the entire volume. Imaging data were preprocessed using SPM12. To correct for head motion, each participant’s data were realigned to the first volume in the time series. The realigned images were spatially normalized into Montreal Neurological Institute stereotactic space using a 12-parameter affine model and smoothed to reduce noise and correct for residual difference in gyral anatomy with a Gaussian filter operationalized at 6-mm full width at half maximum. Voxelwise signal intensities were ratio normalized to the whole-brain global mean. We included all participants with usable data, defined as having fewer than 25% of scans with excessive movement (movement >3 mm of displacement on individual acquisitions) or excessive activation (global signal intensity >10) for a given task. Signal coverage in the regions of interest (ROIs) was excellent for all regions, with at least 80% coverage in the included ROIs. One-sample whole-brain analysis *t* tests for activation in the contrasts of interest across the sample were nonsignificant. This analysis is conservative, however, because we included a majority of youth with personal or family history of depression and/or experience of early adversity. Associated with reduced reward responsivity, these factors could lead to nonsignificant activation across the sample.

**Figure 1:**
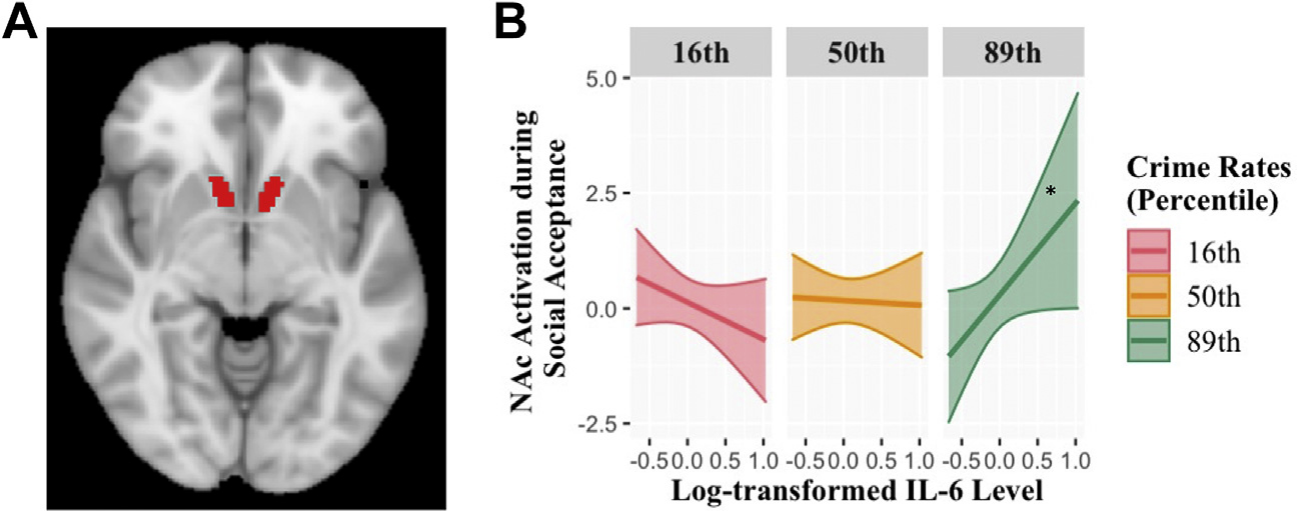
**(A)** Region of interest for the bilateral nucleus accumbens (NAc) defined with Harvard Oxford Atlas template. **(B)** NAc activation during receipt of social acceptance as a function of interleukin (IL) 6 level at the 16th, 50th, and 89th percentiles of neighborhood crime rates. The 89th percentile was selected for illustrating patterns of interaction effect in addition to the 16th and 50th percentiles based on the levels of crime rate at which IL-6 began to be associated with NAc activation. ∗*p* < .05.

**Figure 2:**
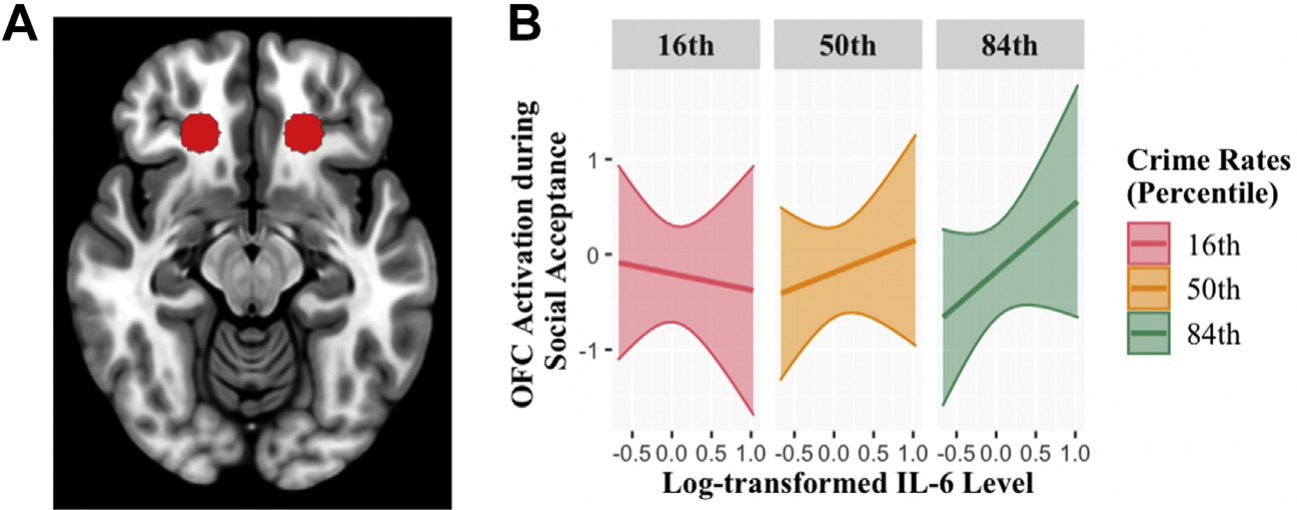
**(A)** Region of interest for the bilateral orbitofrontal cortex (OFC) from a previous meta-analysis (52). **(B)** OFC activation during receipt of social acceptance as a function of interleukin (IL**)** 6 level at the 16th, 50th, and 84th percentiles of neighborhood crime rates. Percentiles were selected for illustrating patterns of (nonsignificant) interaction effect.

A general linear model identified the trial types during anticipation and outcome phases of the card guessing task and the outcome phase of the chatroom task. In the chatroom task, the outcome phase of social acceptance was defined as the period when the social acceptance feedback was presented. In the card guessing task, the reward anticipation phase was defined as the period after the presentation of cues indicating reward trials but before presentation of the actual number. The outcome phase was defined as the period when the actual number and feedback was presented. The last 3000 ms of the intertrial interval was used as a baseline. We included motion covariates in the first-level models to account for activation covarying with blood oxygen level–dependent signal. First-level voxelwise *t* statistics were computed for each participant to produce the following contrasts: monetary reward anticipation > baseline, monetary reward outcome > baseline, and social acceptance outcome > control outcome. Parameter estimates (beta-weights) from predefined ROIs, bilateral NAc (Figure 1A) and OFC (Figure 2A), were extracted and exported into R for analyses. The OFC ROI was defined based on boundaries described in a meta-analysis assessing reward processing abnormalities in depression (52). The bilateral NAc ROI was predefined based on the Harvard Oxford Atlas (53, 54, 55, 56).

### Statistical Analysis

Multivariate linear regression tested whether the main effects of mean-centered IL-6 and neighborhood crime and their interaction were associated with OFC and NAc activation to anticipation/outcome of monetary reward and social acceptance. Analyses controlled a false discovery rate of 5% using the Benjamini–Hochberg procedure for the primary models by reward type (i.e., controlling for 2 tests for social reward and 4 tests for monetary reward) (57). The Johnson-Neyman technique probed the levels of the moderator (i.e., neighborhood crime) at which IL-6 was significantly associated with ROI activation. Body mass index (58,59), current use of psychotropic and anti-inflammatory medication (60), major medical illnesses, depression history, and sex (61) have been associated with inflammation and/or brain function, and analyses were conducted with and without controlling for these variables. To assess specificity to neighborhood crime, analyses were repeated with neighborhood income included as an additional covariate. If results for a given model were significant, supplemental analyses examined the moderating effects of neighborhood income to confirm specificity to neighborhood crime and explored whether a given moderating effect was specific to violent versus nonviolent crime rates.

## Results

Demographics, data characterization, and correlations between study variables are in Tables 1 and 2. Table 3 displays full statistical results for main and interaction effects in primary analyses, with aforementioned covariates and neighborhood income as an additional covariate. The same models are shown in Table S1A, B without inclusion of covariates.

**Table 1:** Summary of Sample Characteristics

Variable	*n* (%) or Mean (SD) [Range]
Sex	
Female	40 (57.14%)
Male	30 (42.86%)
Race	
Bi-/multiracial	2 (2.86%)
Black	43 (61.43%)
White	25 (35.71%)
Psychotropic Medication Status	
Currently taking	15 (21.7%)
Not currently taking	55 (78.3%)
Body Mass Index	24.62 (7.52) [16.47–62.33]
Age at Baseline, Years	13.09 (1.05) [11.17–17.17]
Age at Blood Draw, Years	16.29 (1.64) [12.08–19.92]
Crime Instances During the Year of Study Enrollment	4027.65 (1300.79) [1074–7130]
Annual Household Income in the Neighborhood During the Year of Study Enrollment, $	47,243.73 (13,715.05) [7499.50–95,000]
Interleukin 6, pg/mL	0.59 (0.74) [0.09–5.61]
C-Reactive Protein, mg/L	3.11 (6.58) [0.02–42.86]
Log-Transformed Interleukin 6	1.63 (0.32) [0.95–2.75]
Log-Transformed C-Reactive Protein	1.87 (0.76) [0.26–3.63]

**Table 2:** Correlations Between Main Variables of Interest

Variables	1	2	3	4	5	6	7	8	9	10	11
1 Sex	–	–	–	–	–	–	–	–	–	–	–
2 Body Mass Index	0.140	–	–	–	–	–	–	–	–	–	–
3 Crime	−0.073	−0.134	–	–	–	–	–	–	–	–	–
4 Income	−0.048	−0.22	−0.151	–	–	–	–	–	–	–	–
5 IL-6	0.282^a^	0.395^b^	−0.098	−0.006	–	–	–	–	–	–	–
6 CRP	0.088	0.499^b^	−0.155	−0.054	0.414^b^	–	–	–	–	–	–
7 NAc Social Acceptance	0.176	0.082	−0.045	−0.032	0.082	0.098	–	–	–	–	–
8 OFC Social Acceptance	0.104	−0.153	−0.033	−0.007	0.014	0.061	0.519^b^	–	–	–	–
9 NAc Monetary Reward Anticipation	−0.162	−0.100	−0.054	−0.066	0.048	0.013	<0.001	0.198	–	–	–
10 OFC Monetary Reward Anticipation	−0.168	−0.008	−0.016	−0.133	−0.112	0.022	−0.007	0.201	0.676^b^	–	–
11 NAc Monetary Reward Outcome	0.083	0.016	−0.140	−0.037	0.112	−0.007	0.221	0.200	0.617^b^	0.361^b^	–
12 OFC Monetary Reward Outcome	−0.199	−0.133	−0.038	−0.105	−0.103	−0.173	−0.03	0.190	0.628^b^	0.740^b^	0.532^b^

Correlation results for the association between neural activation to social acceptance and to monetary reward are discussed in the Supplement.

CRP, C-reactive protein; IL-6, interleukin 6; NAc, nucleus accumbens; OFC, orbitofrontal cortex.

a *p* < .05.

b *p* < .01.

**Table 3:** Regression Models of the Relationships Between the Interaction of IL-6 and Neighborhood Crime Rates on NAc and OFC Activation

Dependent Variable	Model 1				Model 2				Model 3			
	*B*	SE	*t*	*p*	*B*	SE	*t*	*p*	*B*	SE	*t*	*p*
NAc: Receipt of Social Acceptance												
Constant	−0.271	0.254	−1.066	.291	−0.197	0.247	−0.799	.428	−0.194	0.250	−0.776	.441
BMI	0.005	0.021	0.241	.810	0.007	0.020	0.346	.730	0.006	0.021	0.283	.778
Sex	−0.368	0.307	1.201	.235	0.325	0.296	1.098	.277	0.322	0.299	1.079	.285
Med	0.107	0.356	0.299	.766	0.081	0.343	0.234	.816	0.078	0.347	0.224	.824
Illness	0.271	0.451	0.602	.550	0.399	0.438	0.911	.367	0.394	0.442	0.892	.376
Dep Hx	0.020	0.318	0.064	.949	0.055	0.308	−0.178	.859	−0.059	0.311	−0.190	.850
SES	–	–	–	–	–	–	–	–	−0.033	0.156	−0.208	.836
IL-6	−0.046	0.542	−0.085	.932	−0.072	0.522	−0.137	.891	−0.057	0.531	−0.107	.915
Crime	<0.001	<0.001	−0.181	.857	<−0.001	<0.001	0.484	.630	<0.001	<0.001	0.454	.652
IL-6 × crime	–	–	–	–	0.001	<0.001	2.342	.023^a^	0.001	<0.001	2.329	.023^a^^*,*^^b^
Δ*R*^2^	–	–	–	–	0.084	–	–	–	0.085	–	–	–
*R*^2^	0.043	–	–	–	0.127	–	–	–	0.128	–	–	–
NAc: Monetary Reward Anticipation												
Constant	0.148	0.117	1.263	.212	0.139	0.119	1.172	.246	0.148	0.119	1.242	.219
BMI	−0.009	0.010	−0.897	.373	−0.009	0.010	−0.919	.362	−0.011	0.010	−1.139	.259
Sex	−0.220	0.140	−1.569	.122	−0.213	0.141	−1.507	.137	−0.218	0.141	−1.540	.129
Med	0.335	0.164	2.043	.046^a^	0.338	0.165	2.047	.045^a^	0.330	0.165	2.001	.050
Illness	−0.117	0.208	−0.561	.577	−0.133	0.211	−0.633	.529	−0.143	0.211	−0.681	.499
Dep Hx	−0.226	0.146	−1.545	.128	−0.217	0.148	−1.469	.147	−0.228	0.148	−1.537	.130
SES	–	–	–	–	–	–	–	–	−0.075	0.074	−1.017	.314
IL-6	0.236	0.249	0.949	.346	0.238	0.250	0.950	.346	0.270	0.252	1.071	.289
Crime	<0.001	<0.001	−0.507	.614	<−0.001	<0.001	−0.676	.502	<0.001	<0.001	−0.801	.426
IL-6 × crime	–	–	–	–	<−0.001	<0.001	−0.615	.541	<−0.001	<0.001	−0.564	.575
ΔR^2^	–	–	–	–	0.005	–	–	–	0.005	–	–	–
*R*^2^	0.160	–	–	–	0.165	–	–	–	0.180	–	–	–
NAc: Monetary Reward Outcome												
Constant	−0.148	0.175	−0.846	.401	−0.122	0.175	−0.694	.491	−0.114	0.177	−0.646	.521
BMI	−0.004	0.014	−0.289	.773	−0.003	0.014	−0.235	.815	−0.006	0.015	−0.372	.711
Sex	0.065	0.209	0.312	.756	0.045	0.209	0.216	.830	0.041	0.210	0.195	.846
Med	0.707	0.244	2.895	.005^c^	0.700	0.244	2.874	.006^c^	0.693	0.245	2.829	.006^c^
Illness	0.125	0.309	0.403	.688	0.174	0.311	0.559	.578	0.165	0.313	0.527	.600
Dep Hx	−0.160	0.218	−0.733	.466	−0.186	0.218	−0.853	.397	−0.195	0.220	−0.888	.378
SES	–	–	–	–	–	–	–	–	−0.066	0.110	−0.598	.552
IL-6	0.144	0.371	0.389	.699	0.140	0.369	0.379	.706	0.168	0.374	0.449	.655
Crime	<−0.001	<0.001	−1.049	.298	<−0.001	<0.001	−0.602	.549	<−0.001	<0.001	−0.671	.505
IL-6 × crime	–	–	–	–	<0.001	<0.001	1.221	.227	<0.001	<0.001	1.243	.219
Δ*R*^2^	–	–	–	–	0.021	–	–	–	0.022	–	–	–
*R*^2^	0.162	–	–	–	0.183	–	–	–	0.188	–	–	–
OFC: Receipt of Social Acceptance												
Constant	−0.039	0.245	−0.158	.875	1.316	0.243	0.054	.957	0.024	0.245	0.100	.921
BMI	−0.025	0.020	−1.222	.227	−0.023	0.020	−1.171	.247	−0.027	0.021	−1.295	.201
Sex	0.288	0.295	0.977	.333	0.258	0.291	0.885	.380	0.249	0.293	0.853	.398
Med	0.398	0.342	1.161	.250	0.379	0.337	1.125	.265	0.370	0.339	1.091	.280
Illness	−0.719	0.434	−1.658	.103	−0.629	0.430	−1.462	.149	−0.642	0.433	−1.485	.143
Dep Hx	0.343	0.305	−1.123	.266	0.396	0.302	−1.309	.196	−0.410	0.305	−1.345	.184
SES	–	–	–	–	–	–	–	–	−0.104	0.153	−0.679	.500
IL-6	0.331	0.521	0.636	.527	0.313	0.513	0.611	.544	0.360	0.520	0.692	.492
Crime	<−0.001	<0.001	−0.264	.793	<0.001	<0.001	0.219	.827	<0.001	<0.001	0.142	.887
IL-6 × crime	–	–	–	–	<0.001	<0.001	1.677	.099	0.001	<0.001	1.695	.096
Δ*R*^2^	–	–	–	–	0.041	–	–	–	0.042	–	–	–
*R*^2^	0.133	–	–	–	0.173	–	–	–	0.180	–	–	–
OFC: Monetary Reward Anticipation												
Constant	0.139	0.082	1.688	.097	0.132	0.083	1.579	.120	0.139	0.083	1.669	.101
BMI	0.004	0.007	0.632	.530	0.004	0.007	0.596	.554	0.002	0.007	0.284	.778
Sex	−0.119	0.099	−1.207	.232	−0.114	0.099	−1.142	.258	−0.117	0.099	−1.186	.241
Med	0.135	0.115	1.168	.247	0.137	0.116	1.182	.242	0.131	0.116	1.132	.262
Illness	−0.129	0.146	−0.884	.380	−0.143	0.148	−0.968	.337	−0.152	0.148	−1.029	.308
Dep Hx	−0.243	0.103	−2.354	.022^a^	−0.235	0.104	−2.261	.028^a^	−0.244	0.104	−2.350	.022^a^
SES	–	–	–	–	–	–	–	–	−0.063	0.052	−1.221	.227
IL-6	−0.146	0.175	−0.833	.408	−0.145	0.176	−0.823	.414	−0.117	0.176	−0.665	.508
Crime	<0.001	<0.001	0.013	.990	<−0.001	<0.001	−0.225	.823	<−0.001	<0.001	−0.381	.704
IL-6 × crime	–	–	–	–	<−0.001	<0.001	−0.734	.466	<−0.001	<0.001	−0.676	.502
Δ*R*^2^	–	–	–	–	0.008	–	–	–	0.007	–	–	–
*R*^2^	0.155	–	–	–	0.163	–	–	–	0.184	–	–	–
OFC: Monetary Reward Outcome												
Constant	0.096	0.101	0.944	.349	0.072	0.100	0.721	.474	0.080	0.100	0.799	.428
BMI	−0.005	0.008	−0.596	.553	−0.006	0.008	−0.697	.488	−0.008	0.008	−0.943	.350
Sex	−0.198	0.121	−1.635	.107	−0.180	0.119	−1.516	.135	−0.184	0.119	−1.553	.126
Med	0.293	0.142	2.066	.043^a^	0.300	0.139	2.165	.035^a^	0.293	0.139	2.118	.039^a^
Illness	−0.007	0.180	−0.039	.969	−0.051	0.177	−0.291	.772	−0.061	0.177	−0.343	.733
Dep Hx	−0.116	0.127	−0.914	.364	−0.091	0.124	−0.739	.463	−0.101	0.124	−0.814	.419
SES	–	–	–	–	–	–	–	–	−0.068	0.062	−1.091	.280
IL-6	−0.063	0.215	−0.294	.770	−0.059	0.210	−0.283	.779	−0.030	0.212	−0.143	.887
Crime	<−0.001	<0.001	−0.629	.532	<−0.001	<0.001	−1.240	.220	<0.001	<0.001	−1.372	.175
IL-6 × crime	–	–	–	–	<−0.001	<0.001	−1.951	.056	<0.001	<0.001	−1.898	.063
Δ*R*^2^	–	–	–	–	0.053	–	–	–	0.050	–	–	–
*R*^2^	0.136	–	–	–	0.189	–	–	–	0.206	–	–	–

The three sets of models present regression models without the interaction term (model 1), with the interaction term (model 2), and with the inclusion of neighborhood SES (model 3). *R*^2^ is the value for the regression model, and Δ*R*^2^ is the change when the IL-6 × crime interaction term is added to the model.

BMI, body mass index; Dep Hx, depression history; FDR, false discovery rate; IL-6, interleukin 6; Med, medication; NAc, nucleus accumbens; OFC, orbitofrontal cortex; SES, socioeconomic status.

a *p* < .05.

b Uncorrected *p* values remained significant after controlling the FDR of 5%.

c *p* < .01.

### Primary Analyses

Main effects for neighborhood crime or IL-6 levels on ROI activation to social acceptance and monetary reward were not significant. As predicted, the interaction between IL-6 and neighborhood crime rates on NAc response to social acceptance was significant (Figure 1B). At the top 10.61% of neighborhood crime rate, elevated IL-6 began to be associated with higher NAc response to social acceptance. We did not find significant interactions between IL-6 and neighborhood crime rates on OFC response to social acceptance (Figure 2B) or on ROI anticipation of or response to monetary reward.

### Secondary Analyses

There was no significant interaction between IL-6 and neighborhood income on neural activation (Table S2). The significant results for NAc social acceptance held for nonviolent, but not violent, neighborhood crime (Table S3A, B). There were no significant associations with CRP (Table S4A, B).

## Discussion

This study examined neighborhood crime rates as a moderator of the relationship between inflammatory signaling and reward-related brain activation. For participants from neighborhoods with higher crime in childhood, higher IL-6 was associated with higher NAc response to social acceptance. However, the association was not significant for those living in low-crime neighborhoods. We also did not find any moderation effect for neighborhood crime rates on the associations of IL-6 with OFC response to social acceptance or ROI responses to monetary reward anticipation or outcome. These patterns were observed before and after adjusting for body mass index, sex, current use of prescription medication, major medical illnesses, depression history, and neighborhood income.

Partially consistent with conceptual models (19,37), the relationship of IL-6 with NAc responses to social acceptance depended on crime rates within participants’ childhood neighborhoods. We detected significant amplification of immune-brain associations for neural response to social acceptance only for those from high-crime neighborhoods. However, we did not observe the predicted amplification effect of neighborhood crime on the crosstalk between inflammation and neural activation to monetary reward. This suggests that the way high crime exposure shapes immune-brain crosstalk may be more nuanced than always taking the form of amplification. This claim is consistent with prior evidence on distinct neurobiological responses and behaviors for individuals with versus without early-life adversity (41,62, 63, 64). Thus, individuals with exposure to high-threat childhood environments might have heightened motivation toward positive social figures and feedback at higher levels of inflammation, which may have evolutionary benefits of optimizing protection from threat to safety. Unexpectedly, we did not find the predicted moderating effect of neighborhood crime on the association between inflammation and neural activation to monetary reward. This may suggest that the moderation effect of high-threat childhood living environments on immune-brain crosstalk is specific to social reward. Alternatively, we may have been underpowered to detect significant effects for responses to monetary reward. Future research should replicate this work using larger sample sizes.

We also did not replicate prior evidence of a significant main effect of inflammation on blunted neural activation to monetary reward and elevated neural activation to social reward (15,22,24, 25, 26). Our study detected a positive association between higher IL-6 and higher NAc activation to social acceptance only among adolescents exposed to neighborhood crime in childhood. Other studies that did not replicate the prior results include that by Miller *et al.* (29), which found a positive correlation between ventral striatal activation during monetary reward anticipation and inflammation only among adolescents living in poverty. Liu *et al.* (18) detected a negative correlation for activation during reward anticipation in the dorsal anterior cingulate cortex but a positive correlation in the NAc in adolescents. Across the aforementioned studies, there is heterogeneity or a lack of report of sample characteristics that may influence immune-brain associations, including developmental stage (e.g., adolescents vs. adults), consideration of prior exposure to childhood adversity in the analyses, health status (e.g., presence of depressive symptoms), and medication use. Considering these sample characteristics in future research not only may reduce noise in analyses but also may offer more comprehensive insight into the neuroimmune processes and their implication for health-related consequences.

Our results support the context-dependence hypothesis that blunted reward-related brain function is not an invariant outcome of inflammation (27,37). Similar to previous studies supporting this hypothesis (24,25,28, 29, 30, 31,37), we showed that the moderating effects of residence in high-crime neighborhoods on immune-brain crosstalk depend on reward type. This highlights a need to carefully delineate the effects of inflammatory signaling on neural responses to reward by teasing apart reward type, in addition to consideration of environmental contexts that may shape neuroimmune connections to help navigate future environments that threaten safety. The traditional view that inflammation regulates neural and behavioral changes in a manner that blunts reward responsiveness may be oversimplistic.

We found a moderating effect for neighborhood crime on the associations between inflammatory signaling and neural social acceptance above and beyond neighborhood income, but not a moderating effect for neighborhood income. This specificity may suggest that the nature of adverse neighborhood characteristics is relevant. This claim also is supported by evidence suggesting that some of the distinct brain structure and function patterns linking childhood adversity to health issues are subject to specific types of adversity (65). Contrary to our null findings on neighborhood income, Miller *et al.* (29) detected an association between inflammation and larger neural reward anticipation among individuals living in poverty. However, the previous study did not measure or adjust for co-occurring neighborhood crime. Thus, it is unclear whether the effects observed in that study were a product of neighborhood crime (29). Future research examining the roles of different forms of childhood adversity in this crosstalk may help distinguish between common versus specific mechanisms underlying different forms of childhood adversities. Our findings imply that the immune-brain mechanisms linking childhood residence in high-crime neighborhoods to psychopathology may be distinct from low neighborhood income.

This study has multiple strengths, including the integration of several developmental models of psychoneuroimmunology (19,27,37) and a comprehensive multimodal framework of analysis that detected effects at the level of an environmental stressor, peripheral immune functioning, and neural activation to social rewards. This study included approximately equal proportions of Black and White adolescents, enhancing generalizability to the Black population, which often is underrepresented in research. Finally, although our analysis was cross-sectional, our dataset was collected prospectively, which allowed us to examine the effects of environmental stress in childhood on adolescents, a developmental group vulnerable to psychopathology.

The findings should be interpreted alongside the study’s limitations. First, absence of longitudinal measurements in our study prevents claims of causality or assessment of the directionality of these relationships. We cannot rule out the plausibility of reverse causality and results driven by a third variable, although our findings are not due to the third variable of neighborhood income. Second, given the heterogeneity of coding crime rates and difficulties in confirming if crime incidence data are standardized across regions, we excluded participants living outside Philadelphia County. Future studies should determine whether these findings are generalizable to other regions. Third, there was no systematic manipulation check on the believability of the deception in the chatroom interact task, limiting the opportunity to check whether any participants’ task performance was biased and how those participants might influence the robustness of the results. However, in previous studies, most participants reported that they believed the deception (47,66, 67, 68). Moreover, several measures were taken to enhance believability (e.g., by showing a fake connection error message followed by an attempt to reconnect and a phone call to the other site to troubleshoot). Fourth, our secondary results with CRP were nonsignificant. Given that CRP also is an inflammatory marker, the results with IL-6 may not be indicative of inflammatory activity. However, our results were consistent with previous research showing that IL-6 more consistently predicts stress levels and depression than CRP (69). Furthermore, there is a lack of consistency in detection of the associations of CRP with reward-related corticostriatal function and psychiatric disorders (e.g., depression) in adolescents (70,71). Fifth, our exploratory analyses suggest that nonviolent crime appeared relevant to the moderating effect on social reward. Future research involving a larger sample is necessary to confirm these findings. Finally, the observed results may be specific to adolescents, a unique group vulnerable for psychopathology with multiple neurobiological systems rapidly changing in parallel. However, we cannot conclude about specificity versus generalizability across the life span without comparing the results to tests involving other age groups. Relatedly, developmental timing of stress exposure has moderated the effects of stress on life span health risk and outcome (64), suggesting that the effect of adverse environmental inputs depends on the developmental period of exposure.

### Conclusion

This study suggests that childhood residence in high-crime neighborhoods amplified the crosstalk between IL-6 and social reward–related brain function in adolescence. This relationship may vary by the social versus nonsocial nature of reward, together offering support of the context-dependent nature of immune-brain crosstalk. In contrast, the traditional view that inflammation-associated behaviors are characterized by blunted reward responsiveness may be oversimplistic. This work may help provide better understanding of the specific role that childhood residence in high-crime neighborhoods plays in the mechanisms underlying multiple health issues during the vulnerable period of adolescence. The findings may identify aspects of health risk for which interventions targeting neighborhood-level crime rates are relevant (19,72). Alternatively, it may be adaptive when high-threat environment exposure informs an increased sensitivity to support others during sickness. We cannot rule out this possibility without actually testing the hypotheses linked to health conditions, although enhanced sensitivity to social stimuli has been linked with psychiatric risk factors [e.g., loneliness and increased risky sexual behavior (73,74)]. Future work should examine whether elevated social reward response is necessarily maladaptive.

## Supplementary Material

Supplementary Material

Key Resources Table

## Footnotes

1 Major medical illnesses included allergy, asthma, cardiovascular disease, a hormonal disorder, a genetic disorder, and chronic pain and migraine from 9 participants.
