# Supplementary material for: Residence in High-Crime Neighborhoods Moderates the Association Between Interleukin 6 and Social and Nonsocial Reward Brain Responses
Source: Biol Psychiatry Glob Open Sci. 2022 May 18;2(3):273–82. doi: 10.1016/j.bpsgos.2022.04.006 (PMC9306340; doi:10.1016/j.bpsgos.2022.04.006)
Supplement: Supplementary Material [file mmc1.pdf]

Residence in High-Crime Neighborhoods Moderates the Association Between Interleukin 6 and Social and Non-Social Reward Brain Responses

Supplemental Information

Additional Description of Measures

Lifetime Depression History. The Kiddie-Schedule for Affective Disorders and Schizophrenia (K-SADS) generates lifetime and current DSM-IV-TR diagnoses for adolescents, as well as clinician-rated symptom scores (1). The interviews were given at baseline and annually throughout a participant's enrollment in the larger, longitudinal Project Adolescent Cognition and Emotion (ACE). K-SADS diagnostic interviews have good inter-rater and retest reliability with a kappa coefficient of .85 in the Project ACE sample based on 120 pairs of ratings (2). Interviewers were blinded to other data collected on the adolescents. An expanded SADS-Lifetime (SADS-L) was completed at annual visits after a participant became 18 years of age or older (3). The SADS-L also yields strong inter-rater reliability with a kappa coefficient of .90 (4). In this sample, any participant who was diagnosed with major depressive disorder or dysthymia based on DSM-IV-TR criteria prior to the date of their fMRI scan were operationalized as having a history of depression.

Neighborhood Crime Rates and Income. Crime incidences were extracted from public Philadelphia Police Department crime statistics, and categorized into violent (e.g., aggravated assault firearm, rape, homicide) and non-violent crimes (e.g., thefts, motor vehicle theft, burglary non-residential) using definition criteria from the United States Bureau of Justice Statistics (5). Participants were matched to their corresponding police district using their street addresses

provided at study enrollment using the police department's "Find My District" website feature. Each participant was given the total number of overall crime, violent crime, and non-violent crime incidents based on the police district in which they resided. Neighborhood income for each police district was calculated by averaging the self-reported income level of all ACE participants (N=492) living in a respective district.

Correlations between neural activation to social acceptance and to monetary reward

Prior studies using fMRI or event-related potentials showed that neural value computations were positively associated for social and monetary reward (6–8). Surprisingly, we did not detect significant correlations between ROI activation to social and monetary reward, which may be attributable to heterogeneity in study designs. Previous studies made the timing and structure of the events similar and matched the number of events of each kind across the social and monetary tasks. The tasks in this study were not matched for the length and number of events within each task. Such differences might have introduced noise to the detection of the associations. Furthermore, because implementation of the Chatroom task required day-of-visit construction of participant specific stimuli (i.e., integrating the participant's pictures into the task), the monetary task was administered first. Thus, we cannot rule out the possibility that order effects may have impacted associations between activation in the tasks.

Supplemental References

1. Orvaschel H (1995): Schedule for Affective Disorders and Schizophrenia for School-Age Children - Epidemiologic Version–5 (K–SADS–E). Nova Southeastern University , Ft. Lauderdale , FL.
2. Alloy LB, Black SK, Young ME, Goldstein KE, Shapero BG, Stange JP, *et al.* (2012): Cognitive Vulnerabilities and Depression Versus Other Psychopathology Symptoms and Diagnoses in Early Adolescence. *Journal of Clinical Child & Adolescent Psychology* 41: 539–560.
3. Endicott J, Spitzer RL (1978): A diagnostic interview: the schedule for affective disorders and schizophrenia. *Arch Gen Psychiatry* 35: 837–844.
4. Alloy L, Abramson L, Hogan M, Whitehouse W, Rose D, Robinson M, *et al.* (2000): The Temple-Wisconsin Cognitive Vulnerability to Depression Project: Lifetime history of Axis I psychopathology in individuals at high and low cognitive risk for depression. *Journal of abnormal psychology* 109: 403–18.
5. Morgan RK (2017): Criminal victimization, 2016. Bureau of Justice Statistics (BJS). *NCJ* 251150.
6. Distefano A, Jackson F, Levinson AR, Infantolino ZP, Jarcho JM, Nelson BD (2018): A comparison of the electrocortical response to monetary and social reward. *Social Cognitive and Affective Neuroscience* 13: 247–255.
7. Wake SJ, Izuma K (2017): A common neural code for social and monetary rewards in the human striatum. *Social Cognitive and Affective Neuroscience* 12: 1558–1564.

8. Rademacher L, Salama A, Gründer G, Spreckelmeyer KN (2014): Differential patterns of nucleus accumbens activation during anticipation of monetary and social reward in young and older adults. *Social Cognitive and Affective Neuroscience* 9: 825–831.

Table S1A. Regression models (without covariates) of the relationships between the interaction of interleukin-6 and neighborhood crime rates on NAc activation

	Model 1				Model 2			
	B	*SE*	*t*	*p*	*B*	*SE*	*t*	*p*
Dependent Variable: NAc Receipt of Social Acceptance								
Constant	0.003	0.141	0.018	0.985	0.039	0.137	0.283	0.778
IL-6	0.270	0.440	0.614	0.541	0.298	0.425	1.702	0.485
Crime	<-0.001	<0.001	-0.292	0.772	<0.001	<0.001	0.309	0.759
IL-6 * Crime	-	-	-	-	0.001	<0.001	2.374	0.021*
ΔR^2					0.083			
R^2	0.008				0.091			
Dependent Variable: NAc Monetary Reward Anticipation								
Constant	0.003	0.069	0.047	0.963	-0.001	0.070	-0.014	0.989
IL-6	0.074	0.216	0.344	0.732	0.068	0.217	0.315	0.754
Crime	<-0.001	<0.001	-0.453	0.652	<-0.001	<0.001	-0.627	0.533
IL-6 * Crime	-	-	-	-	<0.001	<0.001	-0.659	0.512
ΔR^2					0.007			
R^2	0.005				0.012			
Dependent Variable: NAc Monetary Reward Outcome								
Constant	0.002	0.102	0.016	0.987	0.013	0.102	0.126	0.900
IL-6	0.261	0.318	0.820	0.415	0.277	0.318	0.871	0.387
Crime	<-0.001	<0.001	-1.036	0.304	<-0.001	<0.001	-0.636	0.527
IL-6 * Crime	-	-	-	-	<0.001	<0.001	1.188	0.239
ΔR^2					0.021			
R^2	0.029				0.050			

Note. The two sets of models present regression models without the interaction term (Model 1) and with the interaction term (Model 2). Abbreviations: IL-6, interleukin-6; NAc, nucleus accumbens; R^2, r-squared of the regression model; ΔR^2, r-squared change when IL-6 X Crime interaction term is added to the model. * $p < .05$; ** $p < .01$; *** $p < .001$.

Table S1B. Regression models (without covariates) of the relationships between the interaction of interleukin-6 and neighborhood crime rates on OFC activation

	Model 1				Model 2			
	B	*SE*	*t*	*p*	*B*	*SE*	*t*	*p*
Dependent Variable: OFC Receipt of Social Acceptance								
Constant	-0.006	0.143	-0.043	0.966	0.024	0.141	0.167	0.868
IL-6	0.042	0.446	0.095	0.925	0.065	0.438	0.149	0.882
Crime	<-0.001	<0.001	-0.156	0.877	<0.001	<0.001	0.325	0.746
IL-6 * Crime	-	-	-	-	0.001	<0.001	1.891	0.063
ΔR^2					0.054			
R^2	0.001				0.055			
Dependent Variable: OFC Monetary Reward Anticipation								
Constant	0.003	0.048	0.070	0.945	-0.001	0.048	-0.014	0.989
IL-6	-0.140	0.151	-0.925	0.358	-0.145	0.151	-0.961	0.340
Crime	<-0.001	<0.001	-0.320	0.750	<-0.001	<0.001	0.576	0.567
IL-6 * Crime	-	-	-	-	<-0.001	<0.001	-0.905	0.369
ΔR^2					0.013			
R^2	0.014				0.027			
Dependent Variable: OFC Monetary Reward Outcome								
Constant	0.005	0.059	0.090	0.929	-0.005	0.057	-0.091	0.928
IL-6	-0.160	0.183	-0.870	0.387	-0.174	0.179	-0.972	0.335
Crime	<-0.001	<0.001	-0.515	0.608	<-0.001	<0.001	-1.095	0.278
IL-6 * Crime	-	-	-	-	<-0.001	<0.001	-1.981	0.052
ΔR^2					0.058			
R^2	0.015				0.072			

Note. The two sets of models present regression models without the interaction term (Model 1) and with the interaction term (Model 2). Abbreviations: IL-6, interleukin-6; OFC, orbitofrontal cortex; R^2, r-squared of the regression model; ΔR^2, r-squared change when IL-6 X Crime interaction term is added to the model. * $p < .05$; ** $p < .01$; *** $p < .001$.

Table S2. Regression models of the relationships between the interaction of interleukin-6 and neighborhood socioeconomic status on NAc activation.

	Model 1				Model 2				Model 3			
	B	*SE*	*t*	*p*	*B*	*SE*	*t*	*p*	*B*	*SE*	*t*	*p*
Dependent Variable NAc Receipt of Social Acceptance												
Constant	-0.270	0.255	-1.059	0.294	-0.238	0.258	-0.925	0.359	-0.238	0.260	-0.915	0.364
BMI	0.005	0.022	0.228	0.820	0.002	0.022	0.092	0.927	0.002	0.022	0.075	0.941
Sex	0.370	0.306	1.208	0.232	0.342	0.309	1.107	0.273	0.339	0.312	1.087	0.282
Med	0.102	0.356	0.286	0.776	0.021	0.368	0.058	0.954	0.024	0.372	0.066	0.948
Illness	0.279	0.449	0.621	0.537	0.241	0.452	0.533	0.596	0.233	0.458	0.507	0.614
Dep Hx	0.006	0.312	0.018	0.986	0.032	0.314	0.101	0.920	0.042	0.323	0.131	0.896
Crime	-	-	-	-	-	-	-	-	<-0.001	<0.001	-0.164	0.870
IL-6	-0.040	0.547	-0.074	0.942	0.076	0.563	0.134	0.894	0.077	0.568	0.135	0.893
Income	-0.014	0.159	-0.088	0.930	-0.037	0.162	-0.227	0.821	-0.040	0.164	-0.243	0.809
IL-6 * Income	-	-	-	-	-0.445	0.507	-0.877	0.384	-0.442	0.511	-0.864	0.391
ΔR^2					0.013				0.012			
R^2	0.042				0.055				0.056			

Note. The three sets of models present regression models without the interaction term (Model 1), with the interaction term (Model 2), and with the inclusion of neighborhood crime rates (Model 3). Abbreviations: BMI, body mass index; Med, medication; DEP Hx, depression history; IL-6, interleukin-6; NAc, nucleus accumbens; R^2, r-squared of the regression model; ΔR^2, r-squared change when IL-6 X Income interaction term is added to the model. * $p < .05$; ** $p < .01$; *** $p < .001$.

Table S3A. Regression models of the relationships between the interaction of interleukin-6 and neighborhood violent crime rates on NAc activation.

	Model 1				Model 2			
	B	*SE*	*t*	*p*	*B*	*SE*	*t*	*p*
Dependent Variable: NAc Receipt of Social Acceptance								
Constant	-0.269	0.257	-1.046	0.300	-0.229	0.254	-0.904	0.370
BMI	0.005	0.022	0.223	0.825	0.001	0.021	0.050	0.960
Sex	0.363	0.311	1.165	0.249	0.328	0.307	1.069	0.289
Medication	0.109	0.361	0.301	0.765	0.026	0.358	0.073	0.942
Illness	0.271	0.454	0.597	0.553	0.314	0.447	0.703	0.485
Dep Hx	0.020	0.323	0.061	0.951	0.020	0.317	0.063	0.950
Income	-0.031	0.182	-0.169	0.866	-0.032	0.179	-0.179	0.858
IL-6	-0.035	0.552	-0.063	0.950	0.076	0.546	0.139	0.890
Crime	<-0.001	<0.001	-0.195	0.846	<.001	0.001	0.117	0.907
IL-6 * Crime	-	-	-	-	0.003	0.001	1.723	0.091
ΔR^2					0.048			
R^2	0.043				0.091			

Note. The two sets of models present regression models without the interaction term (Model 1) and with the interaction term (Model 2). Abbreviations: BMI, body mass index; DEP Hx, depression history; IL-6, interleukin-6; NAc, nucleus accumbens; R^2, r-squared of the regression model; ΔR^2, r-squared change when IL-6 X Crime interaction term is added to the model. * $p < .05$; ** $p < .01$; *** $p < .001$.

Table S3B. Regression models of the relationships between the interaction of interleukin-6 and neighborhood non-violent crime rates on NAc activation.

	Model 1				Model 2			
	B	*SE*	*t*	*p*	*B*	*SE*	*t*	*p*
Dependent Variable: NAc Receipt of Social Acceptance								
Constant	-0.269	0.257	-1.048	0.299	-0.193	0.250	-0.774	0.442
BMI	0.004	0.022	0.202	0.840	0.001	0.021	0.388	0.699
Sex	0.369	0.309	1.192	0.238	0.328	0.299	1.098	0.277
Medication	0.104	0.359	0.288	0.774	0.111	0.346	0.321	0.750
Illness	0.269	0.455	0.591	0.557	0.407	0.443	0.918	0.363
Dep Hx	0.016	0.320	0.051	0.960	-0.080	0.311	-0.257	0.798
Income	-0.014	0.161	-0.087	0.931	-0.036	0.155	-0.230	0.819
IL-6	-0.039	0.551	-0.071	0.943	-0.117	0.532	-0.220	0.827
Crime	<-0.001	<0.001	-0.180	0.858	<0.001	<0.001	0.501	0.619
IL-6 * Crime	-	-	-	-	0.001	0.001	2.310	0.025*
ΔR^2					0.083			
R^2	0.043				0.126			

Note. The two sets of models present regression models without the interaction term (Model 1) and with the interaction term (Model 2). Abbreviations: BMI, body mass index; DEP Hx, depression history; IL-6, interleukin-6; NAc, nucleus accumbens; R^2, r-squared of the regression model; ΔR^2, r-squared change when IL-6 X Crime interaction term is added to the model. * $p < .05$; ** $p < .01$; *** $p < .001$.

Table S4A. Regression models of the relationships between the interaction of C-reactive protein and neighborhood crime rates on NAc activation.

	Model 1				Model 2				Model 3			
	B	*SE*	*t*	*p*	*B*	*SE*	*t*	*p*	*B*	*SE*	*t*	*p*
Dependent Variable NAc Receipt of Social Acceptance												
Constant	-0.275	0.250	-1.097	0.277	-0.212	0.256	-0.825	0.413	-0.210	0.259	-0.812	0.420
BMI	<0.001	0.021	0.009	0.993	-0.003	0.022	-0.158	0.875	-0.004	0.022	-0.174	0.863
Sex	0.359	0.297	1.1208	0.232	0.325	0.298	1.088	0.281	0.325	0.301	1.078	0.286
Med	0.114	0.355	0.320	0.750	0.093	0.355	0.263	0.793	0.092	0.358	0.257	0.798
Illness	0.238	0.441	0.538	0.592	0.210	0.441	0.475	0.636	0.209	0.445	0.469	0.641
Dep Hx	0.053	0.320	0.165	0.870	0.046	0.320	0.144	0.886	0.043	0.324	0.132	0.895
Income	-	-	-	-	-	-	-	-	-0.018	0.160	-0.113	0.910
CRP	0.105	0.231	0.457	0.649	0.167	0.237	0.704	0.484	0.166	0.239	0.693	0.491
Crime	<-0.001	<0.001	-0.138	0.891	<-0.001	<0.001	-0.159	0.875	<0.001	<0.001	-0.170	0.865
CRP * Crime	-	-	-	-	<0.001	<0.001	1.087	0.282	<0.001	<0.001	1.078	0.286
ΔR^2					0.019				0.019			
R^2	0.046				0.065				0.066			
Dependent Variable NAc Monetary Reward Anticipation												
Constant	0.125	0.116	1.074	0.287	0.120	0.120	0.996	0.323	0.125	0.120	1.035	0.305
BMI	-0.007	0.010	-0.663	0.510	-0.006	0.010	-0.621	0.537	-0.008	0.010	-0.769	0.445
Sex	-0.190	0.137	-1.383	0.172	-0.187	0.139	-1.342	0.185	-0.187	0.140	-1.340	0.186
Med	0.351	0.165	2.130	0.037*	0.353	0.166	2.120	0.038*	0.347	0.167	2.084	0.042*
Illness	-0.079	0.205	-0.386	0.701	-0.077	0.207	-0.372	0.711	-0.081	0.207	-0.389	0.699
Dep Hx	-0.234	0.149	-1.574	0.121	-0.234	0.150	-1.558	0.125	-0.246	0.151	-1.627	0.109
Income	-	-	-	-	-	-	-	-	-0.066	0.074	-0.893	0.376
CRP	0.037	0.107	0.346	0.731	0.032	0.111	0.288	0.775	0.029	0.112	0.256	0.799
Crime	<-0.001	<0.001	-0.445	0.658	0.000	0.000	-0.441	0.661	<0.001	<0.001	-0.569	0.572
CRP * Crime	-	-	-	-	<-0.001	<0.001	-0.189	0.851	<-0.001	<0.001	-0.179	0.859
ΔR^2					0.001				<0.001			
R^2	0.148				0.149				0.161			
Dependent Variable NAc Monetary Reward Outcome												
Constant	-0.156	0.172	-0.909	0.367	-0.154	0.178	-0.865	0.391	-0.150	0.179	-0.835	0.407
BMI	0.000	0.015	0.026	0.979	0.000	0.015	0.016	0.987	-0.001	0.015	-0.071	0.943
Sex	0.085	0.203	0.419	0.677	0.084	0.206	0.406	0.686	0.084	0.208	0.403	0.688
Med	0.710	0.244	2.911	0.005**	0.709	0.246	2.880	0.006**	0.705	0.248	2.842	0.006**
Illness	0.168	0.303	0.553	0.583	0.167	0.306	0.544	0.589	0.164	0.308	0.530	0.598
Dep Hx	-0.187	0.220	-0.850	0.399	-0.188	0.222	-0.844	0.402	-0.197	0.225	-0.878	0.383

Income	-	-	-	-	-	-	-	-	-0.055	0.111	-0.492	0.624
CRP	-0.060	0.159	-0.376	0.708	-0.057	0.165	-0.347	0.730	-0.060	0.166	-0.362	0.719
Crime	<-0.001	<0.001	-1.067	0.290	<-0.001	<0.001	-1.058	0.294	<-0.001	<0.001	-1.113	0.270
CRP * Crime	-	-	-	-	<0.001	<0.001	0.061	0.951	<0.001	<0.001	0.066	0.948
ΔR^2					<0.001				<0.001			
R^2	0.162				0.162				0.166			

Note. The three sets of models present regression models without the interaction term (Model 1), with the interaction term (Model 2), and with the inclusion of neighborhood income (Model 3). Abbreviations: BMI, body mass index; Med, medication; DEP Hx, depression history; CRP, C-reactive protein; NAc, nucleus accumbens; R^2, r-squared of the regression model; ΔR^2, r-squared change when CRP X Crime interaction term is added to the model. * $p < .05$; ** $p < .01$; *** $p < .001$.

Table S4B. Regression models of the relationships between the interaction of C-reactive protein and neighborhood crime rates on OFC activation.

	Model 1				Model 2				Model 3			
	B	*SE*	*t*	*p*	*B*	*SE*	*t*	*p*	*B*	*SE*	*t*	*p*
Dependent Variable OFC Receipt of Social Acceptance												
Constant	-0.087	0.239	-0.363	0.718	-0.015	0.243	-0.061	0.951	-0.010	0.245	-0.041	0.968
BMI	-0.031	0.020	-1.508	0.137	-0.035	0.021	-1.699	0.095	-0.037	0.021	-1.746	0.086
Sex	0.325	0.283	1.148	0.256	0.286	0.283	1.011	0.316	0.286	0.285	1.001	0.321
Med	0.439	0.339	1.298	0.199	0.417	0.337	1.236	0.222	0.411	0.339	1.212	0.231
Illness	-0.721	0.421	-1.713	0.092	-0.753	0.419	-1.796	0.078	-0.756	0.422	-1.791	0.079
Dep Hx	-0.293	0.306	-0.959	0.342	-0.301	0.304	-0.991	0.326	-0.314	0.307	-1.022	0.311
Income	-	-	-	-	-	-	-	-	-0.073	0.152	-0.484	0.630
CRP	0.278	0.220	1.266	0.211	0.348	0.225	1.547	0.127	0.345	0.227	1.520	0.134
Crime	<-0.001	<0.001	-0.145	0.885	<-0.001	<0.001	-0.170	0.866	<-0.001	<0.001	-0.229	0.820
CRP * Crime	-	-	-	-	<0.001	<0.001	1.303	0.198	<0.001	<0.001	1.297	0.200
ΔR^2					0.025				0.025			
R^2	0.150				0.175				0.178			
Dependent Variable OFC Monetary Reward Anticipation												
Constant	0.152	0.082	1.863	0.067	0.166	0.084	1.971	0.054	0.171	0.084	2.046	0.045*
BMI	0.002	0.007	0.312	0.756	0.001	0.007	0.197	0.844	0.000	0.007	-0.046	0.964
Sex	-0.138	0.097	-1.430	0.158	-0.146	0.098	-1.496	0.140	-0.146	0.097	-1.507	0.137
Med	0.127	0.116	1.094	0.279	0.123	0.117	1.052	0.297	0.117	0.116	1.012	0.316
Illness	-0.157	0.144	-1.093	0.279	-0.163	0.145	-1.126	0.265	-0.167	0.144	-1.161	0.250
Dep Hx	-0.232	0.105	-2.212	0.031*	-0.233	0.105	-2.216	0.031*	-0.246	0.105	-2.343	0.023*
Income	-	-	-	-	-	-	-	-	-0.070	0.052	-1.358	0.180
CRP	-0.002	0.075	-0.027	0.979	0.012	0.078	0.148	0.883	0.008	0.077	0.102	0.919
Crime	<-0.001	<0.001	-0.013	0.990	<-0.001	<0.001	-0.016	0.988	<-0.001	<0.001	-0.220	0.827
CRP * Crime	-	-	-	-	<0.001	<0.001	0.726	0.471	<0.001	<0.001	0.745	0.459
ΔR^2					0.008				0.008			
R^2	0.145				0.153				0.179			
Dependent Variable OFC Monetary Reward Outcome												
Constant	0.109	0.099	1.104	0.274	0.099	0.102	0.967	0.337	0.104	0.102	1.028	0.308
BMI	-0.002	0.008	-0.181	0.857	-0.001	0.009	-0.110	0.913	-0.003	0.009	-0.328	0.744
Sex	-0.204	0.117	-1.750	0.085	-0.198	0.118	-1.677	0.099	-0.198	0.118	-1.684	0.098
Med	0.280	0.140	2.002	0.050*	0.283	0.141	2.008	0.049*	0.277	0.141	1.973	0.053
Illness	0.007	0.174	0.042	0.966	0.012	0.175	0.067	0.947	0.007	0.175	0.042	0.967
Dep Hx	-0.141	0.126	-1.115	0.270	-0.140	0.127	-1.099	0.276	-0.154	0.127	-1.209	0.232

Income	-	-	-	-	-	-	-	-	-0.078	0.063	-1.240	0.220
CRP	-0.111	0.091	-1.217	0.229	-0.121	0.094	-1.282	0.205	-0.125	0.094	-1.331	0.189
Crime	<-0.001	<0.001	-0.748	0.458	<-0.001	<0.001	-0.741	0.462	<-0.001	<0.001	-0.922	0.360
CRP * Crime	-	-	-	-	<-0.001	<0.001	-0.453	0.652	<-0.001	<0.001	-0.442	0.661
ΔR^2					0.003				0.003			
R^2	0.156				0.159				0.181			

Note. The three sets of models present regression models without the interaction term (Model 1), with the interaction term (Model 2), and with the inclusion of neighborhood income (Model 3). Abbreviations: ROI, region of interest; Med, medication; DEP Hx, depression history; CRP, C-reactive protein; OFC, orbitofrontal cortex; R^2, r-squared of the regression model; ΔR^2, r-squared change when CRP X Crime interaction term is added to the model. * $p < .05$; ** p < .01; *** $p < .001$.
